# Bacteremia in Severe Mastitis of Dairy Cows

**DOI:** 10.3390/microorganisms11071639

**Published:** 2023-06-23

**Authors:** Isabel Krebs, Yanchao Zhang, Nicole Wente, Stefanie Leimbach, Volker Krömker

**Affiliations:** 1 Department of Bioprocess Engineering, Microbiology, Faculty II, Hannover University of Applied Sciences and Arts, 30453 Hannover, Germany; isabel.krebs@tiho-hannover.de (I.K.); yanchao.zhang@hs-hannover.de (Y.Z.); nicole.wente@hs-hannover.de (N.W.); stefanie.leimbach@hs-hannover.de (S.L.); 2 Department of Veterinary and Animal Sciences, University of Copenhagen, 1870 Frederiksberg C, Denmark

**Keywords:** severe mastitis, dairy cow, bacteremia, blood culture, pathogen shedding

## Abstract

The aim of this cross-sectional study was to investigate the occurrence of bacteremia in severe mastitis cases of dairy cows. Milk and corresponding blood samples of 77 cases of severe mastitis were bacteriologically examined. All samples (milk and blood) were incubated aerobically and anaerobically to also investigate the role of obligate anaerobic microorganisms in addition to aerobic microorganisms in severe mastitis. Bacteremia occurred if identical bacterial strains were isolated from milk and blood samples of the same case. In addition, pathogen shedding was examined, and the data of animals and weather were collected to determine associated factors for the occurrence of bacteremia in severe mastitis. If Gram-negative bacteria were detected in milk samples, a Limulus test (detection of endotoxins) was also performed for corresponding blood samples without the growth of Gram-negative bacteria. In 74 cases (96.1%), microbial growth was detected in aerobically incubated milk samples. The most-frequently isolated bacteria in milk samples were *Escherichia* (*E*.) *coli* (48.9%), *Streptococcus* (*S*.) spp. (18.1%), and *Klebsiella* (*K*.) spp. (16%). Obligatory anaerobic microorganisms were not isolated. In 72 cases (93.5%) of the aerobically examined blood samples, microbial growth was detected. The most-frequently isolated pathogens in blood samples were non-*aureus* Staphylococci (NaS) (40.6%) and *Bacillus* spp. (12.3%). The Limulus test was positive for 60.5% of cases, which means a detection of endotoxins in most blood samples without the growth of Gram-negative bacteria. Bacteremia was confirmed in 12 cases (15.5%) for *K*. *pneumoniae* (5/12), *E. coli* (4/12), *S. dysgalactiae* (2/12), and *S. uberis* (1/12). The mortality rate (deceased or culled) was 66.6% for cases with bacteremia and 34.1% for cases without bacteremia. High pathogen shedding and high humidity were associated with the occurrence of bacteremia in severe mastitis.

## 1. Introduction

Mastitis is one of the most-frequent diseases on dairy farms [1]. In addition to the negative impacts on animal welfare and udder health, this disease causes high economic losses on dairy farms and is a main reason for culling [2]. Furthermore, following chronic damage, such as declining milk yield, high somatic cell counts (SCCs), and decreased fertility often occur after mastitis [2,3,4].

According to the definitions of the International Dairy Federation (IDF) (2011) [5], severe mastitis is characterized by abnormalities in the milk, local signs of inflammation of the udder, and symptoms of a systemic disease, such as abnormalities in body temperature and behavior. Affected animals with severe mastitis can show inappetence, apathy, and an inability to stand upright. Schmenger and Krömker (2020) [6] showed a prevalence of severe mastitis of 9.1%. Fredebeul-Krein et al. (2022) [7] described a prevalence of 21.1% for severe mastitis. High mortality rates of 13.5% [8] to 35% [9] for severe mastitis were described.

Bacteremia is defined as the presence of bacteria in the blood, which can only be diagnosed by a microbiological finding. In contrast, sepsis is a clinical diagnosis and is characterized by severely disturbed general well-being. Sepsis can result from bacteremia, but also from other pathogens (e.g., viruses, fungi) or toxins circulating in the blood [10,11]. The blood–milk barrier (BMB) prevents an uncontrolled exchange of components between blood and milk. Udder tissue damage occurs during mastitis. This results in a disturbed BMB, which suggests the passage of bacteria from milk into the blood [12]. Most bacteremia cases do not develop into sepsis because the bacteria are cleared from the bloodstream before causing systemic damage [13]. The occurrence of bacteria in the blood causes oxygen release from erythrocytes. The released oxygen and humoral immunity of the host kill bacteria in the bloodstream. If bacteria are immune to oxygen and proliferate in erythrocytes, sepsis occurs [13].

To treat severe mastitis and bacteremia, antibiotic therapy of mastitis appears to be unavoidable. In general, it is recommended to always treat severe mastitis parenterally with antibiotics because of the risk of bacteremia and the high risk of losing animals [9,14,15,16]. The effect of parenteral antibiotics in mastitis therapy is not only limited to the infected udder quarter, but also affects commensal bacteria throughout the body. Therefore, parenteral antibiotics promote antimicrobial resistance because of the effect on the microbiome and should only be used if bacteria have spread systemically. For this reason, parenteral antibiotics are only justified in cases of bacteremia associated with bacteria-induced mastitis. However, in many cases of severe mastitis, no pathogens are detected in the udder secretion. In studies with the same culture methods, the rate of no microbial isolation in milk from severe mastitis ranges from 24.2% [14] to 30.5% [6]. A frequent reason for no microbial isolation of mastitis in milk samples is an insufficient bacterial count, which cannot be detected with usual examination methods [17]. A study reported that 34.9% of cases treated with intramammary antibiotics showed no bacterial growth [18]. In milk production, most antibiotic use is due to mastitis therapy and prevention [19]. In contrast, as a reaction to the currently increasing occurrence of antimicrobial resistance, a significant reduction in the use of antibiotics in livestock production is required [20].

For the diagnosis of bacteremia linked to mastitis, a detection of identical bacteria in milk and blood is necessary. Overall, there are only a few studies on the prevalence and related pathogens in bacteremia in dairy cows. The prevalence of bacteremia in dairy cows ranged from 1.4% [14] to 32% [9]. Brennecke et al. (2021) [14] carried out a previous study in which bacteremia was already proven for *E. coli*. Wenz et al. (2001) [9] showed that bacteremia is also associated with infections of *Klebsiella* spp. However, it is difficult to determine the exact prevalence of bacteremia. Although blood cultures are considered the gold standard for diagnosing bacteremia in human medicine, there is a high risk of contamination, especially through the patient’s skin [21]. In veterinary medicine, contamination from the animal’s fur makes diagnostics even more difficult.

Little is known about the factors influencing bacteremia in dairy cows. The number of lactations and days in milk were already investigated for the occurrence of bacteremia, but no association was determined [9]. The following factors are positively correlated with the occurrence of severe mastitis: pathogen shedding [7], milk production [7,22], previous treatments with corticosteroids [7], and previous occurrence of mastitis and other diseases [7,23]. Oliveira et al. (2013) [22] found no association between previous mastitis and the severity of the current mastitis. SCC before mastitis was examined as a risk factor for CM in several studies. Dairy cows with low SCC are associated with a higher incidence of CM [24,25], which can also suggest a relation to severe mastitis. Environmental factors also influence the severity of CM. Severe mastitis occurs more frequently in the summer months compared to moderate and mild mastitis [7]. Milk fat content was also examined in relation to the severity of CM, with low milk fat content being associated with severe mastitis rather than with mild and moderate mastitis [7].

The aim of this study was to determine the prevalence and the associated factors of bacteremia in severe mastitis to research more about the occurrence of bacteremia. In a previous study, bacteremia in dairy cows was already examined [14]. In the present study, a timely transport after sample collection and a short transport time is an important point due to an improvement in the detection of bacteria in the blood. Another focus is to investigate which mastitis pathogens can trigger bacteremia and are frequently involved in it. As blood cultures are aerobically and anaerobically examined in human medicine [26], this study also examined the importance of anaerobes in severe mastitis in dairy cows. In addition, the therapy of severe mastitis is discussed.

## 2. Materials and Methods

All applicable guidelines for the care and use of animals were followed. The study was approved by the animal welfare committee of the university (University of Veterinary Medicine Hanover, Foundation; file reference: TVO-2021-V-51). The date when ethical approval was obtained was the fifteenth of July 2021. An application for a license for animal testing was not required by the local government due to the study design. The study complied with the International Guiding Principles for Biomedical Research Involving Animals (1985).

### 2.1. Study Design

This cross-sectional study was carried out in the period from July 2021 to August 2022. Milk and blood samples from cows with severe mastitis were collected from 13 dairy farms in Lower Saxony, Germany. The farms participated in a dairy herd improvement (DHI) program. Selection criteria were an especially large herd size for the highest possible number of samples from severe mastitis and a close location to the laboratory for a short sample transport. Most dairy farms had a herd size of more than 500 cows, but one farm with a close location and small herd size was also included. The herd size ranged from 170 to 2500 Holstein Frisian cows with an average 305 d milk yield of 11,500 kg. The average SCCs of the bulk tank ranged between 150,000 and 250,000 cells/mL. The cows of all herds obtained a total mixed ration and were milked two or three times a day in a side-by-side parlor, herringbone parlor, rotary milking parlor, or by a milking robot. The animals were kept in free-stall barns with cubicles or deep litter barns with compost or straw.

The personnel received training from the veterinarian for recognizing cases of severe mastitis and performing correct sample collection in accordance with the IDF standard (2011) [5] and the guidelines of the Society of Veterinary Medicine in Germany (2018) [27]. One symptom of a disturbed general condition according to GVA (2009) [28], e.g., fever, was sufficient for the case to be included in the study as a severe case. During the milking process, farm personnel detected severe mastitis cases and collected milk samples from each udder quarter of the affected animal. A veterinarian took a blood sample from affected cows and treated the animals immediately, for example with non-steroidal anti-inflammatory drugs (NSAIDs), antibiotics, and fluid. The samples were transported to the microbiological laboratory of the University of Applied Sciences and Arts Hannover (Hannover, Germany) within 48 h for analysis. Milk and blood samples were aerobically and anaerobically examined. If the milk sample and the blood sample from one cow contained the same pathogen species, strain typing was performed. In the case of an identical strain, bacteremia was assumed. If Gram-negative microorganisms were found in the milk sample, a Limulus test was performed on the corresponding blood samples. No growth of Gram-negative bacteria in these blood samples was required for conducting the Limulus test. A positive result of the Limulus test can suggest a higher prevalence of bacteremia than the microbiological findings show. To determine the exact number of pathogens, the grown colonies were counted quantitatively at different dilution levels. In addition, animal-specific and environmentally related data were collected for each case.

### 2.2. Sampling

#### 2.2.1. Milk Samples

In case of severe mastitis, the milking personnel immediately collected milk samples from all quarters before antimicrobial treatment in accordance with the guidelines of GVA (2009) [28]. Before collection, the apex of each teat was cleaned and disinfected with 70% ethanol. After discarding the first three streams of milk, the samples were taken aseptically. Ten milliliters of milk were collected. Sample tubes containing boric acid (Ly20) as a preserving agent were used [29]. During sampling, disposable gloves were worn. Milk samples were stored at 6 °C until they were transported to the laboratory.

#### 2.2.2. Blood Samples

Blood samples were taken from the jugular vein by a veterinarian directly before antimicrobial treatment. Therefore, animals’ skin was disinfected with 70% ethanol three times, and two 20 mL samples of blood were collected using two 20 mL syringes with 18-gauge needles. Then, each of the samples was directly injected through a new 18-gauge needle into one of our own injection bottles containing 80 mL of Brain Heart Infusion Broth (Merck KgaA, Darmstadt, Germany) and 0.025% sodium polyanetholsulfonate (anticoagulant) (Merck KgaA) through the rubber stopper. The blood culture bottles were produced in the same laboratory of microbiological examination. Commercial blood cultures are only available for small pets, which are not designed for the examined blood volume in this study. To produce the blood culture bottles, we followed the instructions from Neumeister et al. (2009) [26]. A blood- to-media ratio of 1 to 5 is required, and a brain heart broth with an anticoagulant is most suitable for blood cultures. To reduce the risk of contamination, all outer rubber stopper surfaces were disinfected with 70% ethanol before injecting the blood sample. The blood samples were stored at 37 °C until being transported to the laboratory.

### 2.3. Laboratory Procedures

#### 2.3.1. Milk Samples

Milk samples were examined in accordance with the GVA (2018) [27] guidelines, which are similar to the National Mastitis Council (NMC) procedures (NMC) (1999) [30].

A total of 100 µL of each sample was plated in duplicate using serial dilution (10^−1^ to 10^−4^) on esculin blood agar (Oxoid Deutschland GmbH, Wesel, Germany). Only the agar plates with 10 to 300 colonies were considered for the calculation of colony-forming units per mL (cfu/mL). This resulted in a lower detection limit of 100 cfu/mL. Due to the highest number of countable colonies of 300 colonies per plate, the upper detection level was 3 × 10^6^ cfu/mL. One set was incubated aerobically and the other one anaerobically at 37 °C. The aerobically incubated plates were analyzed after 24 h and 48 h. Plates to be examined on obligate anaerobes were incubated for 7 d under anaerobic conditions. The grown colonies were counted quantitatively for each plate and pathogen. Subsequently, the total pathogen shedding (cfu/mL) was calculated for each pathogen using the number of colonies.

The grown colonies were differentiated by means of Gram staining, morphology and cell morphology, hemolysis patterns, and esculin hydrolysis. Gram-positive catalase positive cocci (3% H_2_O_2_, Merck KGaA, Darmstadt, Germany) were identified as NaS. A clumping factor test (DiaMondiaL Staph Plus Kit, Sekisui Virotech GmbH, Russelsheim, Germany) was used in β-hemolyzing staphylococci to discriminate between *Staphylococcus aureus* and NaS. Catalase-negative Gram-positive esculin hydrolyzing cocci were cultured on a modified Rambach agar medium to differentiate *Enterococcus* (*E*.) spp. and *S. uberis*. Esculin non-hydrolyzing streptococci were classified according to the Lancefield serotyping by using the Strep Latex Kit (DiaMondiaL, Vienna, Austria).

Catalase-negative Gram-positive irregular rods with a Y-shaped cell configuration and β-hemolysis were identified as *Trueperella* (*T*.) *pyogenes*. Gram-positive, non-hemolytic catalase-positive irregular rods were defined as coryneforms. Yeasts and *Prototheca* spp. were determined by microscopy. Gram-negative rods were differentiated by their ability to catabolize glucose under aerobic and anaerobic conditions (glucose supplemented oxidation–fermentation test medium, Merck KgaA) and cytochrome C oxidase production (Bactident Oxidase, Merck KgaA). Cytochrome-C-oxidase-negative colonies fermenting glucose were cultured on Chromocult^®^Coliform Agar (Merck KgaA) to distinguish *E. coli* and other coliforms. Non-motile coliforms were determined as *Klebsiella* spp. Gram-negative, cytochrome-C-oxidase-positive bacteria, which metabolized glucose oxidatively, were identified as *Pseudomonas* spp. Samples were considered as contaminated if more than two different pathogen species were detected. The SCC of the milk samples was determined by flow cytometry (SomaScope Smart^TM^, PerkinElmer LAS (Germany) GmbH, Rodgau, Germany).

#### 2.3.2. Blood Samples

For the anaerobic examination, blood cultures were filled up with sterile paraffin oil until no air was visible in the vials. The incubation time for anaerobic culture was 7 d and for aerobic culture 48 h. All blood cultures were immediately incubated after sampling at 37 °C. The incubation time until the transport to the laboratory was included in the total incubation time. The blood cultures were transported at room temperature. After incubation, all blood cultures were plated on esculin blood agar. The further sample processing was identical to the milk sample procedures. All obtained isolates were stored at −80 °C in Brain Heart Infusion Broth with 20% glycerol.

#### 2.3.3. Strain Comparison

Matrix-assisted laser desorption time-of-flight (MALDI TOF) analysis (Bruker Daltonics, microflex LT/SH smart, MBT Compass Library, V8) was performed to confirm the species of the obtained isolates. Only the species of isolates on which the bacteriological examination suggested a match were confirmed by MALDI TOF. All matching species from the entire samples of the same case were compared by randomly amplified polymorphic deoxyribonucleic acid (DNA) polymerase chain reaction (RAPD PCR).

For DNA extraction DNeasy Blood and Tissue Kit (Qiagen Benelux B.V., Venlo, The Netherlands) was used. The reaction mix volume was 25 μL based on 12.5 μL of ReadyMix™ Taq PCR Reaction Mix (Sigma-Aldrich Chemie GmbH, Munich, Germany), 20 pmol of primer (Table 1), 5 µL of the template, and pure PCR-grade water. The PCR was carried out in a LifeTouch Thermocycler (Hangzhou Bioer Technology Co., Ltd., Hangzhou, China).

All PCR products were stained (MIDORIGreen Direct, NIPPON Genetics Europe GmbH, Düren, Germany), separated on a 2% agarose gel, and visualized with the software GeneSnap (Syngene International Ltd., Cambridge, U.K.). Identical RAPD patterns of the PCR products were defined as the same strain. Table 1 contains the applied primers for the RAPD PCR. The amplification conditions for Primer OPE04 were 1× 94.5 °C for 120 s and 35 × 94.5 °C for 70 s, 33 °C for 60 s, and 72 °C for 130 s. The amplification conditions for Primer ERIC-1R were 1× 2 min at 94 °C, 35× 1 min at 94 °C, 1 min at 25 °C, 4 min at 72 °C and 1 × 1 min at 94 °C, 1 min at 25 °C, and 8 min at 72 °C.

#### 2.3.4. Limulus Test

The Limulus test directly detects the endotoxins of Gram-negative bacteria. The test was only carried out on blood samples in which no Gram-negative bacteria were culturally detected, but were detected in the corresponding milk sample. The test was carried out using the ToxinSensor™ Gel Clot Endotoxin Assay Kit (GenScript USA Inc., Piscataway, NJ, USA). The limulus amoebocyte lysate contains a gel-forming protein from blood cells of the Horseshoe crab (Limulus polyphemus), which reacts with the endotoxins of Gram-negative bacteria. Blood samples were considered as positive if the viscosity of the mixture increased. The minimal detection limit of the endotoxin level occurred at 0.25 EU/mL. As this test can only detect endotoxins, a positive result of the blood samples was not defined as bacteremia, but showed an infiltration of lipopolysaccharides into the blood.

### 2.4. Data Collection

Table 2 contains data representing possible influencing factors and their categorization for the occurrence of severe mastitis and bacteremia collected for each case. The cow-associated data were collected from the herd management program and DHI records. Weather data were collected from the nearest weather station to the farm.

### 2.5. Statistical Analysis

The collection and processing of the data were carried out with Microsoft Excel 2021 (Microsoft Corp., Redmond, WA, USA). To analyze the dataset, the program SPSS 28.0, IBM, Inc., Chicago, IL, USA, was used. The udder quarter with a severe CM case was the statistical unit. Associations between bacteremia of occurring mastitis cases and potential risk factors (independent variables) were examined with generalized linear mixed models with logit link and binomial response (bacteremia y/n (logistic regression)) after pre-screening for variable selection in univariable analysis.

The relation between dependent and independent variables was tested first with appropriate univariable tests. Multicollinearity was checked with Spearman/Kendall’s tau, which indicated a correlation of r > 0.70 with one another. For this reason, no variables were excluded. Then, independent variables associated with the dependent variable at *p* < 0.10 in the univariable test were submitted to generalized linear mixed models. Using logistic regression procedures, the association between bacteremia and risk factors (independent variables) was examined. Herd was considered as a random effect.

A backward stepwise procedure was used to select the final multivariable regression model. Potential risk factors were excluded if *p* > 0.05.

Meaningful biological interactions between the fixed effects were also used in the final model if significant (*p* < 0.05) and if they did not increase the Akaike information criterion (AIC). Non-significant effects or interactions that increased the AIC were not included in the final model. Model fit was evaluated by checking the normality of the residuals.

Odds ratios (ORs) were calculated to describe the direction of the relationship between dependent and independent variables. The ORs were determined with 95% confidence intervals (CI 95%), and statistical significance was set at *p* ≤ 0.05. For statistical analysis, the calculated number of pathogens was logarithmized (log10 cfu/mL) to obtain a normal distribution. The THI was used as a covariate.

## 3. Results

### 3.1. Descriptive Results

#### 3.1.1. Milk Samples

A total of 77 cases of severe mastitis were examined. In 74 cases (96.1%), microbial growth was detected in aerobically incubated milk samples. Mixed infections (two different pathogens) occurred in 12 of 77 cases. In addition, 5 of 77 dairy cows had two quarters with mastitis. This resulted in a total of 82 bacteriologic findings (Table 3). *E. coli* (47.6%, 39/82), *Streptococcus* spp. (13.4%, 11/82), and *Klebsiella* spp. (*K. pneumoniae* and *K. oxytoca*) (10.9%, 9/82) were the most-frequently isolated pathogens. The pathogen distribution in anaerobically incubated milk inoculum was similar to that of the aerobe examination. In three cases of aerobically incubated and in one case of anaerobically incubated milk inoculum, more than two different pathogens were detected, which were considered as contaminated. Despite the anaerobic incubation, obligate anaerobes were not isolated from the milk samples. Other isolated pathogens were, for example, *T. pyogenes*, coryneforms, and *Proteus* spp. The microbiological findings from the neighboring quarters were only considered if the same bacterial species was found in the associated blood sample.

#### 3.1.2. Blood Cultures

In 72 of 77 (93.5%) cases of the aerobically examined blood samples, microbial growth was detected. A total of 27 aerobically incubated blood samples were contaminated. All in all, 29 blood samples showed a mixed growth of two different pathogens, so there was a total of 106 findings (Table 4).

In 75 of 77 (97.4%) cases of the anaerobically examined blood samples, microbial growth was detected, 10 blood samples were contaminated, and 31 blood samples showed a mixed growth, so there was a total of 108 findings (Table 4).

The most-frequently isolated pathogens from the blood samples were NaS (40.6%, 43/106) and *Bacillus* spp. (12.3%, 13/106) (Table 4). In anaerobic blood cultures, *Enterococcus* spp. (*E. faecalis* and *E. faecium*) were frequently isolated pathogens (13.9%, 15/108). In contrast to the results from the milk samples, obligate anaerobes (*Clostridium* spp.) were isolated (2.9%, 3/106). Other isolated pathogens were, for example, *Enterobacter* spp. and *Cutibacterium acnes*. All isolates of the contaminated samples were identified so that a strain comparison could be carried out in case of matching bacteria. The contaminated blood samples mostly showed a growth of NaS and *Bacillus* spp.

#### 3.1.3. Bacteremia

Contaminated blood samples were included in the study if pathogen species matched in the blood and milk sample. In 17 of 77 cases, the pathogen species from the milk sample and the associated blood culture matched (Table 5). The strain comparison showed that, in 12 matching cases, the species had identical RAPD patterns. Therefore, a bacteremia rate of 15.5% (12/77) was determined in this study. In detail, bacteremia was detected for *K. pneumoniae* (5/12), *E. coli* (4/12), *S. dysgalactiae* (2/12), and *S. uberis* (1/12). In the remaining five cases, no identical RAPD patterns could be found; all these cases were caused by *E. coli*. In four of five cases with two quarters with mastitis, bacteremia was proven (Table 5). In one case of a mixed infection with matching *E. coli* and *K. pneumoniae*, only *K. pneumoniae* was identical. In two cases of bacteremia, the identical pathogens (*S. dysgalactiae, S. uberis*) were isolated from neighboring quarters and not from the quarter with mastitis. Five cases of bacteremia were only detectable because of the anaerobic examinations of the samples.

#### 3.1.4. Results for Collected Data

Most cases of bacteremia had pathogen shedding of over 3 × 10^6^ cfu/mL (75%, 9/12). In contrast, only 32.3% (20/62) of cases without proven bacteremia showed pathogen shedding of over 3 × 10^6^ cfu/mL (Table 6).

Most cases of bacteremia occurred with a relative humidity of over 80% (Table 7). Cases without bacteremia occurred independently of the relative humidity.

Data from the subsequent three months after mastitis were collected from 53 cases, 43 cases without bacteremia and 10 cases with bacteremia (Table 8). Most affected animals (51%, 27/53) survived, of which bacteremia was confirmed in 3 cases (*E. coli*, *S. uberis*, *S. dysgalactiae*). The mortality rate for all cases was 39.6% (21/53). The mortality rate for cases with bacteremia was 66.6% (6/9). The causative bacteria in these fatal cases of bacteremia were *E. coli* (3/6) and *K*. *pneumoniae* (3/6). In 9.4% (5/53) of cases, a mastitis-independent reason was given for leaving the dairy farms. A total of 34.1% (15/44) of cows without proven bacteremia were culled due to mastitis.

Dairy cows with a *Klebsiella* spp. infection mostly died due to mastitis. A total of 75% (6/8) of cases with *Klebsiella* spp. found in milk samples died (deceased due to this mastitis); of these were 3 cases with bacteremia and two affected quarters. The mortality rate for cases with *E. coli* isolates in milk was much lower. A total of 35.5% (11/31) of affected cows with *E. coli* mastitis died (Table 9).

Most severe mastitis cases occurred in dairy cows that were at least in their second lactation (93.2%, 69/74) and their first third of lactation (47.3%, 35/74). A total of 52.9% (33/70) of all cases showed no abnormality in body temperature (fever or undertemperature) during mastitis. In most cases, the average SCC of the last three DHI reports was lower than 100,000 cells/mL (58.3%, 42/72). A total of 50% (29/58) of cases showed an average milk yield of 10,000 to 12,000 kg per lactation and a daily milk yield of over 40 kg (56.9%, 37/65). Most cases showed in the last DHI report a milk protein content of 3 to 3.5% (70%, 49/70) and a milk fat content of 3 to 4% (62.9%, 44/70). Most affected animals with severe mastitis had no previous illnesses (62.3%, 43/69) and had not received any previous treatment in the previous 30 days (80.8%, 59/73). With a small majority, most cases occurred at a temperature of 15 °C to 20 °C (35.1%, 26/74). Most samples were taken in the summer months (July through September) (75.3%, 58/77). The total number of data varied between the variables due to a different number of missing data.

#### 3.1.5. Limulus Test

The Limulus test was performed in 43 cases. The test was positive for 26 cases (60.5%) and negative for 17 cases (39.5%) (Table 10).

### 3.2. Results of Final Generalized Linear Mixed Models

Associations between bacteremia and potential risk factors were examined with generalized linear mixed models. Two risk factors were significantly associated with the occurrence of bacteremia in severe mastitis (Table 11). The pathogen shedding was significantly associated with the occurrence of bacteremia (*p* = 0.036). The risk of developing bacteremia in severe mastitis increased the higher the pathogen shedding was (OR 1.866, CI 1.04–3.34). In addition, the relative humidity showed a significant association with the occurrence of bacteremia (*p* = 0.049). The likelihood of developing bacteremia increased with a high relative humidity (OR 1.052, CI 1–1.106). The statistical analysis showed no association between THI and bacteremia occurrence.

## 4. Discussion

Despite bacteremia in dairy cows with mastitis being an extensive and important issue, only a few studies exist [9,14,34,35]. In the present study, a bacteremia rate of 15.5% was detected. The Limulus test was positive in most cases, meaning that endotoxins appeared in the blood. The source of these endotoxins cannot be determined by this test. However, it is possible that the endotoxins originated from Gram-negative bacteria that caused mastitis in the udder, but could not be microbiologically detected in the blood. Therefore, positive results can suggest a higher prevalence of (non-detected) bacteremia than the microbiological examinations showed. Brennecke et al. (2021) [14] reported a bacteremia rate of 1.4%, while Wenz et al. (2001) [9] detected a rate of 32%. Many reasons can be responsible for the different results. As blood cultures for dairy cows are not commercially available, they were prepared in the laboratory. Blood clots worsen the detection rate of bacteremia, so adding an anticoagulant is recommended. Most common is the use of 0.025–0.05% sodium polyanetholsulfonate. In human medicine, at least 10 mL of blood is taken for a blood culture [26]. Since it is known that a larger amount of blood also means a higher chance of detecting bacteremia, we decided to use a blood volume of 20 mL [36]. The number of samples taken is another important factor. Taking multiple samples increases the likelihood of detecting bacteremia [37]. Unfortunately, we were not able to take several blood samples at different time points due to the immediate intravenous therapy with antibiotics. Due to the low level of resistance in veterinary medicine, it would be unlikely to detect bacteremia in the blood samples taken after antibiotic therapy. In human medicine, it is usual to investigate the blood for anaerobes [26]. In our study, obligate anaerobes seem to be of little importance for severe mastitis, but without the examination for anaerobes, we would have discovered significantly fewer cases of bacteremia. On the one hand, the total amount of blood and the number of samples doubled because of the examination for anaerobes. On the other hand, some strains of *E. coli* and *Klebsiella* spp. also grow under anaerobic conditions. In addition, it is difficult to take the blood sample at the exact time point. In many cases of bacteremia, an intermittent inflow of bacteria into the bloodstream is common [38,39]. Nevertheless, we decided against taking samples at different times because the sampled animals were mostly treated with antibiotics intravenously after the blood sample had been taken. The decision to use antibiotics was farm dependent. Taking blood samples after antibiotic therapy could falsify the results of blood cultures [26]. In commercially available blood cultures for humans and pets, there are substances (e.g., resin or glass beads) that can neutralize antibiotics through previous antibiotic therapy to rule out false negative results [26]. This point could be considered in future studies where blood samples are taken at different times.

The sensitivity of our blood cultures was very high, but the results in our study indicated frequent contamination. *Bacillus* spp. and NaS are the most-common contaminants of blood cultures and were also mostly isolated in the blood samples of our study [34,35,40]. The contaminated blood samples with more than two different isolates also mostly showed the growth of NaS and *Bacillus* spp. The main cause of contamination in human medicine is the patient’s skin [21,40]. Stable conditions and the fur of the animal make it more difficult to take samples aseptically. In the study by Wenz et al. (2001) [9], the skin was shaved to prevent contamination. However, blood sampling from a dairy cow with a severely disturbed general condition must be performed quickly. We decided against shaving because of the stress involved for the animal and the considerable amount of time required for this task. Another source of contamination could be the hands of the person taking the samples. Despite hand disinfection or changing gloves, contamination is possible. The source of other bacteria detected in blood cultures without proving bacteremia due to mastitis is unclear. These bacteria can be contaminants or real bacteremia due to other bacterial herds. Frequent growth of *Enterococcus* spp. in the anaerobic blood cultures was detected. In human medicine, enterococcal bacteremia is quite common and associated with a high mortality rate for immunosuppressed patients [41]. In that study, enterococcal bacteremia were caused mostly by *E. faecalis* and *E. faecium*, which is consistent with our results for *Enterococcus* spp. It is possible that enterococcal bacteremia also exists in dairy cows. One possible source could be the gastrointestinal tract.

Two streptococcal bacteremias were only detected because of the bacterial findings in neighboring quarters. The streptococci found in the blood were identical to those found in neighboring quarters without CM. In these cases, the mastitis quarter was caused by other bacteria than streptococci, which suggests a contamination of the blood cultures.

The bacteriologic examination of the blood cultures revealed five matching *E. coli* in the blood and associated milk samples, but the strains were not identical. One possible explanation could be that the mastitis was caused by different *E. coli* strains that were morphologically indistinguishable in the bacteriologic examination of the samples. A human medicine study showed no difference between the virulence factors of *E. coli* isolates in blood and a local herd of infection [42]. Therefore, the assumption that *E. coli* strains are unable to enter the bloodstream due to their microbiological characteristics is rather unlikely. Another explanation could be that the *E. coli* strains in the blood came from the gastrointestinal tract or were due to other infections. Contamination of the blood samples from the hands of the personnel or the fur of the animal is also possible. Contamination, however, would not explain the fact that *E. coli* isolates were only detected in blood samples of cases with *E. coli* isolates in milk.

In addition, a positive correlation of severe mastitis with humidity and pathogen shedding was determined. Hamel et al. (2021) [43] showed increasing pathogen shedding for high THI values. The THI is a parameter that is often used to evaluate heat stress in dairy cows [43]. In humid climates, the relative humidity is the limiting factor of heat stress [33]. In our study, no association between the occurrence of bacteremia and outside temperature and THI was detected. Most bacteremia occurs in the summer months, but low temperatures with high humidity also promote bacteremia. Cows use transpiration for thermoregulation. Therefore, they suffer from heat stress especially when the relative humidity is high [33]. The more difficult thermoregulation in high humidity could be an explanation for these results.

Bacteremia can only develop if the integrity of the BMB is reduced. It is known that pathogens can damage the BMB [12,44]. Therefore, high pathogen shedding could be associated with a higher risk of a destroyed BMB, which allows the bacteria to enter the bloodstream.

The mortality rate for bacteremia cases was much higher than for cases without bacteremia. Wenz et al. (2001) [9] described a negative impact of infections with *E. coli* and *K. pneumoniae* on survival. A total of 35% of those cases died, which agrees with our results for the mortality rate of *E. coli* infections (35.5%). However, the mortality rate for *Klebsiella* spp. infections was much higher. Cha et al. (2013) [45] found that an infection in the first lactation with *Klebsiella* spp. is associated with a higher risk of mortality than an infection with *E. coli*. *Klebsiella*-spp.-induced mastitis could be underestimated because it occurred less frequently than *E. coli* or streptococci. This study clearly showed that, although *Klebsiella*-spp.-induced mastitis occurred less frequently, it had a higher mortality rate and a high risk of developing bacteremia. Bacteremia was only proven for *K. pneumoniae*. Further studies are needed to determine the role of *K. oxytoca* in bacteremia. Most cases with two quarters with mastitis were cases with severe mastitis caused by *K. pneumoniae*. The high mortality and bacteremia rate can also be a result of infecting two quarters. Two quarters with mastitis increase the bacterial shedding, which means a higher risk of destroyed BMB. However, further studies are needed to reinforce these hypotheses. In human medicine, every additional hour after the development of sepsis without treatment increases mortality by 6% [13]. Therefore, the time of detection of severe mastitis is crucial for the prognosis. Most severe cases were caused by *E. coli*. In the literature, *Streptococcus* spp., followed by *E. coli*, were the most-common causes of severe mastitis [7,14]. The results also depend on the dairy farms because each farm has a different microbiota. In general, streptococci and *E. coli* are particularly common causes of severe mastitis.

Antibiotics have become an integral part of mastitis therapy. Systemic antibiotic therapy is even recommended for severe mastitis cases because there is a risk of developing bacteremia [9,15,16]. Before answering the question about whether systemic antibiotics are necessary in severe mastitis, the prevalence and pathogenesis of bacteremia, as well as the effectiveness of systemic antibiotics must be clarified. A study about the effectiveness of systemic ceftiofur in severe cases of mastitis showed a higher survival rate of treated animals with coliform mastitis [8]. However, there was no difference in the mortality rate between treated and untreated severe cases regardless of the causative bacteria. Thus, a systemic therapy with antibiotics of coliform mastitis could be necessary. On the other hand, many studies showed high bacteriologic cure rates of mastitis caused by coliform mastitis [6]. The severity classification of mastitis is, therefore, an important point in the therapy decision. Suojala et al. (2013) [16] described the importance of parenteral antibiotic therapy in severe *E. coli* mastitis, but in mild and moderate mastitis cases, a therapy with NSAID and fluid is sufficient. An important fact is that not every case of bacteremia ends in sepsis. In bacteremia, two events are critical for the development of sepsis: infection resistance to oxidation and intensive release of oxygen to arterial blood. Sepsis can only be diagnosed based on clinical symptoms and culture-based pathogen detection [13]. In our study, a prevalence of 15.5% bacteremia was established, which means in 15.5% of cases, a parenteral therapy of antibiotics was indicated. Nonetheless, an overestimate of the bacteremia rate is possible due to the high risk of contamination. On the other hand, an underestimate is also conceivable due to the risk of missing the timepoint at which the bacteremia is detectable. The progressive development of bacterial resistance complicates a therapy decision in these cases. However, withholding medical care for these 15.5% cases of bacteremia is ethically unacceptable for animal welfare reasons. Further studies with a larger number of cases are needed for a better understanding of bacteremia in dairy cows.

## 5. Conclusions

A total of 15.5% bacteremia cases were detected. The Limulus test detected endotoxins in most blood samples (60.5%), which could indicate a higher prevalence of bacteremia. Most bacteremia cases were caused by *K. pneumoniae*. *E. coli* caused the most-severe mastitis cases. A positive correlation of severe mastitis with pathogen shedding and relative humidity was determined. Parenteral antibiotic therapy is indicated in bacteremia cases due to animal welfare reasons, especially in severe cases of mastitis caused by *Klebsiella* spp. and *E. coli* due to a high mortality rate.

## Figures and Tables

**Table 1:** Applied primers for RAPD PCR ^1^.

Pathogen	Primer, Sequence	Reference
Streptococci	OPE 04 5′-GTGACATGCC-3′	Gillespie et al., 1998 [31]
Coliforms, NaS ^2^	ERIC 1R 5′-ATGTAAGCTCCTGGGGATTCAC-3′	Vogel et al., 1999 [32]

^1^ Randomly amplified polymorphic deoxyribonucleic acid polymerase chain reaction. ^2^ Non-aureus staphylococci.

**Table 2:** Potential risk factors of severe mastitis and bacteremia at quarter, animal, and herd level.

Risk Factor	Description	Categories
Body temperature	Body temperature at the time of illness in degrees Celsius	Abnormality (<38.0 °C, ≥39.5 °C) and normal (38.0–39.4 °C)
Number of lactations	Number of lactations on which mastitis appeared	1 and >1
Days in milk	Day of lactation on which the disease appeared	1. third (<100 d), 2. third (100–200 d), 3. third (>200 d)
Average SCC ^1^	Average SCC ^1^ calculated from SCCs ^1^ of the last 3 months in thousand cells per milliliter	≤100, 101–500, 501–1000, >1000
Average 305 d milk yield	Average 305 d milk yield in kilograms calculated from all lactations if completed	<10,000 10,000–12,000 >12,000
Daily average milk yield	Daily average milk yield in kilograms in the current lactation	<30, 30–40, >40
Protein content	Last protein content of the milk in percent	<3, 3–3.5, >3.5
Fat content	Last fat content of the milk in percent	<3, 3–4, >4
Previous illnesses	All previous diseases of the affected dairy cow in the current lactation	None, mastitis, claw disease, fertility disease
Pretreatments	All treatments in the last 30 days with an indication of the active substance	None, antibiotics, NSAID ^2^
Outcome	Follow-up data of the affected cow for the next 3 months after the day of illness	Survived, Died (deceased, euthanized, culled due to this mastitis), Other (culled or left the farm due to reasons other than the present mastitis)
Outside temperature	Outside temperature measured on the day of illness at 12 o’clock in degrees Celsius	<15 15–20 21–25 >25
Temperature–humidity index (THI) ^3^	Value calculated from outside temperature and humidity	No categorization

^1^ Somatic cell count. ^2^ Non-steroidal anti-inflammatory drugs. ^3^ THI was calculated based on the following equation [33]: THI = (1.8 × T + 32) − (0.55 − 0.0055 × RH) × (1.8 × T − 26); (T = outside temperature (°C), RH = relative humidity (%)).

**Table 3:** Isolated pathogens in milk samples of quarters with mastitis.

Bacteriological Findings	*n*	%
*Escherichia coli*	39	47.6
*Streptococcus* spp.	11	13.4
*Klebsiella* spp.	9	10.9
Other	4	4.9
NaS ^1^	1	1.2
Mixed infections	12	14.6
No growth	3	3.7
Contaminated	3	3.7
Total	82 ^2^	100

^1^ Non-*aureus* staphylococci. ^2^ Five cases with two quarters of mastitis.

**Table 4:** Isolated pathogens in aerobically incubated blood inocula.

Isolated Pathogens in Blood	*n*	%
NaS ^1^	43	40.6
*Bacillus* spp.	13	12.3
*Acinetobacter* spp.	8	7.6
*Escherichia coli*	1	0.9
*Klebsiella pneumoniae*	2	1.9
Other	3	2.8
*Enterococcus* spp.	3	2.8
*Streptococcus* spp.	1	0.9
No growth	5	4.7
Contaminated	27	25.5
Total	106 ^2^	100

^1^ Non-*aureus* staphylococci. ^2^ Twenty-nine mixed growths.

**Table 5:** Matching bacteria and cases of bacteremia associated with the number of quarters with mastitis.

Pathogens	Matching Cases	Cases of Bacteremia	Cases with Two Quarters with Mastitis	Pathogens Isolated from Quarter with Mastitis
*Klebsiella pneumoniae*	5	5	3	5
*Escherichia coli*	9	4	0	9
*Streptococcus dysgalactiae*	2	2	1	1
*Streptococcus uberis*	1	1	0	0
Total	17	12	4	15

**Table 6:** Pathogen shedding in association with the occurrence of bacteremia.

Bacteremia	<100,000 cfu/mL	100,000–3,000,000 cfu/mL	>3,000,000 cfu/mL	Total
Yes	1	2	9	12
No	23	19	20	62
Total	24	21	29	74 ^1^

^1^ Three contaminated milk samples.

**Table 7:** Relative humidity in association with the occurrence of bacteremia.

Bacteremia	<60%	60–70%	71–80%	>80%	Total
Yes	1	2	3	6	12
No	18	10	21	13	62
Total	19	12	24	19	74 ^1^

^1^ Three contaminated milk samples.

**Table 8:** Outcome and mortality rate of cases with and without bacteremia.

Bacteremia	Died ^1^	Survived	Other ^2^	Total Cases with Data
Yes	6	3	0	9
No	15	24	5	44

^1^ Deceased, euthanized, or culled due to this mastitis. ^2^ Culled or left the farm due to reasons other than mastitis.

**Table 9:** Follow-up data and mortality rate in association with pathogen findings in milk samples.

Pathogen	Died ^1^	Survived	Other ^2^	Total
*Klebsiella* spp.	6	2	0	8
*E. coli*	10	17	4	31
*Streptococcus* spp.	2	7	0	8
NaS ^3^	0	3	0	3
Other	1	2	0	3
No growth	2	0	0	2
Contaminated	2	0	1	3
Total	23	31	5	59 ^4^

^1^ Deceased, euthanized, or culled due to this mastitis. ^2^ Culled or left the farm due to reasons other than mastitis. ^3^ Non-*aureus* staphylococci. ^4^ Six mixed infections.

**Table 10:** Results of Limulus test in blood in association with pathogen findings in milk samples.

Pathogen	Positive	Negative
*Escherichia coli*	18	12
*Klebsiella* spp.	4	1
Other	3	3
Contaminated	1	1
Total	26	17

**Table 11:** Generalized linear mixed model for the relationship between risk factors and bacteremia in severe mastitis.

Risk Factor	Regression Coefficient	Standard Error	*t*-Value	*p*-Value	OR ^2^	95% CI ^3^
Intercept	−8.846	2.8711	−3.081	0.003	0.000	4.729 × 10^−7^–0.044
Pathogen shedding ^1^ (logarithmized)	0.624	0.2927	2.132	0.036	1.866	1.04–3.34
Relative humidity	0.051	0.0253	2.002	0.049	1.052	1–1.106

^1^ Logarithmized. ^2^ Odds ratio. ^3^ Ninety-five percent confidence interval for odds ratio.

## Data Availability

The data presented in this study are available upon request from the corresponding author. The data are not publicly available due to privacy.
